# Population Pharmacokinetics of the Antimalarial Amodiaquine: a Pooled Analysis To Optimize Dosing

**DOI:** 10.1128/AAC.02193-17

**Published:** 2018-09-24

**Authors:** Ali Mohamed Ali, Melissa A. Penny, Thomas A. Smith, Lesley Workman, Philip Sasi, George O. Adjei, Francesca Aweeka, Jean-René Kiechel, Vincent Jullien, Marcus J. Rijken, Rose McGready, Julia Mwesigwa, Kim Kristensen, Kasia Stepniewska, Joel Tarning, Karen I. Barnes, Paolo Denti, for the WWARN Amodiaquine PK Study Group, Achille Massougbodji

**Affiliations:** a Swiss Tropical and Public Health Institute, Basel, Switzerland; b University of Basel, Basel, Switzerland; c Ifakara Health Institute, Bagamoyo, Tanzania; d Division of Clinical Pharmacology, Department of Medicine, University of Cape Town, Cape Town, South Africa; e WorldWide Antimalarial Resistance Network (WWARN) Clinical Pharmacology and Southern African Regional Hub, University of Cape Town, Cape Town, South Africa; f Muhimbili University of Health and Allied Sciences, Dar-es-Salaam, Tanzania; g Centre for Tropical Clinical Pharmacology and Therapeutics, College of Health Sciences, University of Ghana, Korle Bu, Ghana; h Office of Research, Innovation and Development, University of Ghana, Legon, Ghana; i Department of Clinical Pharmacy, School of Pharmacy, University of California, San Francisco, California, USA; j Drugs for Neglected Diseases Initiative, Geneva, Switzerland; k Service de Pharmacologie, Hôpital Européen George Pompidou, Université Paris Descartes, Paris, France; l Shoklo Malaria Research Unit, Mahidol-Oxford Tropical Medicine Research Unit, Faculty of Tropical Medicine, Mahidol University, Mae Sot, Thailand; m Centre for Tropical Medicine and Global Health, Nuffield Department of Medicine, University of Oxford, Oxford, United Kingdom; n Medical Research Council Unit, Fajara, the Gambia; o Faculty of Medicine and Health Sciences, University of Antwerp, Antwerp, Belgium; p Development DMPK-PKPD, Novo Nordisk, Copenhagen, Denmark; q Worldwide Antimalarial Resistance Network, University of Oxford, Oxford, United Kingdom; r Mahidol-Oxford Tropical Medicine Research Unit, Faculty of Tropical Medicine, Mahidol University, Bangkok, Thailand

**Keywords:** NONMEM, dose optimization, malaria, pediatrics

## Abstract

Amodiaquine plus artesunate is the recommended antimalarial treatment in many countries where malaria is endemic. However, pediatric doses are largely based on a linear extrapolation from adult doses. We pooled data from previously published studies on the pharmacokinetics of amodiaquine, to optimize the dose across all age groups. Adults and children with uncomplicated malaria received daily weight-based doses of amodiaquine or artesunate-amodiaquine over 3 days. Plasma concentration-time profiles for both the parent drug and the metabolite were characterized using nonlinear mixed-effects modeling. Amodiaquine pharmacokinetics were adequately described by a two-compartment disposition model, with first-order elimination leading to the formation of desethylamodiaquine, which was best described by a three-compartment disposition model. Body size and age were the main covariates affecting amodiaquine clearance. After adjusting for the effect of weight, clearance rates for amodiaquine and desethylamodiaquine reached 50% of adult maturation at 2.8 months (95% confidence interval [CI], 1.5 to 3.7 months) and 3.9 months (95% CI, 2.6 to 5.3 months) after birth, assuming that the baby was born at term. Bioavailability was 22.4% (95% CI, 15.6 to 31.9%) lower at the start of treatment than during convalescence, which suggests a malaria disease effect. Neither the drug formulation nor the hemoglobin concentration had an effect on any pharmacokinetic parameters. Results from simulations showed that current manufacturer dosing recommendations resulted in low desethylamodiaquine exposure in patients weighing 8 kg, 15 to 17 kg, 33 to 35 kg, and >62 kg compared to that in a typical 50-kg patient. We propose possible optimized dosing regimens to achieve similar drug exposures among all age groups, which require further validation.

## Abstract (precis)

Amodiaquine plus artesunate is the recommended antimalarial treatment in many countries where malaria is endemic. However, pediatric doses are largely based on a linear extrapolation from adult doses.

## INTRODUCTION

Approximately 3.2 billion people were at risk of Plasmodium falciparum malaria in 2015, with an estimated 212 million malaria cases and 429,000 malaria-related deaths occurring that year. Children under 5 years of age carry the highest burden of disease, accounting for 70% of malaria-related deaths in 2015 (1). Artemisinin resistance has been confirmed in at least six countries in Southeast Asia (2–4). Thus, both the choice of and correct dosing of artemisinin-based combination therapies (ACTs) are crucial and should be driven by a comprehensive understanding of the pharmacokinetic (PK) and pharmacodynamic (PD) properties of both drugs in the combination.

Artesunate plus amodiaquine is one of the five ACTs currently recommended by the World Health Organization (WHO) and has been adopted as a first-line or second-line treatment in at least 25 countries (5). Four different amodiaquine-based treatments are available, including amodiaquine alone, coblistered amodiaquine plus artesunate (loose combination), an amodiaquine-plus-artesunate fixed-dose combination, and coblistered amodiaquine plus sulfadoxine-pyrimethamine. A recent meta-analysis showed that amodiaquine plus artesunate administered in a fixed-dose formulation results in efficacy higher than that of the other formulations (6).

Amodiaquine is primarily metabolized in the liver by cytochrome P450 (CYP) 2C8 (CYP2C8) to its biologically active metabolite, desethylamodiaquine (7–9), which is thought to be the driver of the antimalarial activity (8, 10). Desethylamodiaquine is eliminated slowly and has been detected in plasma and blood for up to 1 month after drug administration (10). Desethylamodiaquine is further metabolized *in vivo*, via an unknown route, into its inactive metabolite, bis-desethylamodiaquine (11). Both amodiaquine and desethylamodiaquine are over 90% protein bound (12) to α_1_-acid glycoprotein (13).

Several studies have investigated dosing regimens for current antimalarial combination therapies (14–19). There is some concern that dosing recommendations may not be optimal for some subgroups of patients, such as young and/or malnourished children and pregnant women, due to altered PK properties in these vulnerable groups. Several studies have investigated the PK of amodiaquine and desethylamodiaquine using both model-based and model-independent analyses (20–25). However, to the best of our knowledge, no studies have investigated the population PK of amodiaquine and desethylamodiaquine across a broad range of ages by combining individual patient-level data from different studies. Pooling of data from different populations allows for the assessment of clinically important determinants affecting PK parameters that might not be possible to investigate in smaller individual studies, and the large amount of data provides more accurate and reliable parameter estimates.

The objective of this analysis was to investigate the PK of amodiaquine and desethylamodiaquine by pooling data from previously published studies, using nonlinear mixed-effects modeling. The developed model was used to assess the current dosing recommendations and to propose an improved dosing regimen, if necessary.

(This analysis was part of Ali Mohamed Ali's Ph.D. work, so the information in this article will be included in his Ph.D. dissertation.).

## RESULTS

Thirteen relevant clinical studies were identified, of which eight were shared with the WorldWide Antimalarial Resistance Network (WWARN). Two studies were contributed after the database for analysis was locked and were therefore not included. Data from 261 patients were available for PK analysis. Of these patients, 95 (36.4%) were children under 5 years of age and 26 were pregnant women (21, 24). The median age at the baseline across the studies was 7.6 years (interquartile range [IQR], 3.6 to 18 years; range, 1 to 60 years). All patients were infected with Plasmodium falciparum, except for those in one study cohort from Asia (21, 24), which had Plasmodium vivax monoinfections. The geometric mean baseline parasitemia was 24,400 parasites/μl of blood (IQR, 7,880 to 76.500 parasites/μl of blood; range, 240 to 566,000 parasites/μl of blood) and 114 parasites/μl of blood (IQR, 288 to 3,920 parasites/μl of blood; range, 96 to 50,500 parasites/μl of blood) in patients infected with Plasmodium falciparum and Plasmodium vivax, respectively. Baseline values for each study population are presented in Table 1, and detailed information can be found in the individually published reports (20–25). Children from Burkina Faso (who were among the youngest) received a higher total dosage (median, 33.8 mg/kg of body weight; range, 14.8 to 65.6 mg/kg) than the other study populations (Kruskal-Wallis test, *P* < 0.001) (Table 1).

**TABLE 1:** Patient characteristics at baseline^*a*^

Characteristic	Value(s) for the following study(ies):					
	Tarning and colleagues (21, 24)	Jullien et al. (23)	Mwesigwa et al. (25)	Stepniewska et al. (22)	Adjei et al. (20)	Total
No. of patients	26 (+7)^*b*^	53	20	61	101	261
% of male patients (no. of male patients/total no. of patients)	0 (0/26)	47.2 (25/53)	65.0 (13/20)	54.1 (33/61)	50.5 (51/101)	46.7 (122/261)
Median (range) age (yr)	23.0 (16.0–39.0)	24.0 (18.0–60.0)	9.0 (6.0–13.0)	2.5 (1.0–5.0)	6.0 (1.0–14.0)	7.6 (1.0–60.0)
% of patients aged (yr) (no. of patients of the indicated age/total no. of patients):						
<2	0 (0/26)	0 (0/53)	0 (0/20)	32.8 (20/61)	12.9 (13/101)	12.6 (33/261)
2 to <5	0 (0/26)	0 (0/53)	0 (0/20)	65.6 (40/61)	21.8 (22/101)	23.8 (62/261)
5 to <12	0 (0/26)	0 (0/53)	90.0 (18/20)	1.6 (1/61)	53.5 (54/101)	28.0 (73/261)
12+	100 (26/26)	100 (53/53)	10.0 (2/20)	0 (0/61)	11.9 (12/101)	34.6 (93/261)
% of patients receiving the following drug formulation, treatment regimen (no. of patients receiving the formulation, regimen/total no.):						
AQ + AS, FDC	0 (0/26)	47 (25/53)	0 (0/20)	47.5 (29/61)	0 (0/101)	20.7 (54/261)
AQ + AS, separate tablets	0 (0/26)	53 (28/53)	100 (20/20)	52.5 (32/61)	85.2 (86/101)	63.6 (166/261)
AQ alone	100 (26/26)	0 (0/53)	0 (0/20)	0 (0/61)	14.8 (15/101)	15.7 (41/261)
Enrollment demographic, vital, and laboratory parameters						
Median (range) wt (kg)	49.0 (37.0–68.0)	59.0 (39.0–90.0)	24.5 (20.0–42.0)	12.5 (7.0–31.0)	18.0 (6.5–93.0)	21.0 (6.5–93.0)
Median (range) total dose (mg/kg)	30.5 (28.1–63.0)	29.5 (18.0–47.1)	24.3 (23.9–26.0)	33.7 (14.8–65.6)	30.0 (30.0–30.0)	30.0 (14.8–65.6)
Geometric mean (range) parasitemia (no. of parasites/μl)	1,142 (96–50,453)^*c*^	11,923 (1,127–109,356)	11,122 (240–174,800)	23,110 (1,357–467,600)	42,689 (630–566,358)	17,952 (96–566,358)
Median (range) hematocrit (%)	32.5 (23.0–40.0)					32.5 (23.0–40.0)
Median (range) hemoglobin concn (g/dl)	10.3 (6.7–13.2)^*d*^	13.2 (9.9–17.7)	12.2 (9.7–14.1)	8.7 (5.9–12.4)	11.6 (6.5–15.1)	11.2 (5.9–17.7)

a Percentages can be more than 100% due to rounding errors. AQ, amodiaquine; AS, artesunate; FDC, fixed-dose combination.

b Seven patients were sampled again after delivery, during another episode of malaria.

c The data are for patients with vivax malaria.

d Derived on the basis of their hematocrit value.

A total of 2,920 postdose venous plasma samples for both amodiaquine and desethylamodiaquine were collected, but the majority of patients (162/261; 62.1%) contributed only 1 to 3 samples. Three studies (147 patients) collected samples for drug measurements before the first study dose, but only 1 (0.7%) amodiaquine concentration and 29 (19.7%) desethylamodiaquine concentrations were measured to be above the lower limit of quantification (LLOQ) (Table 2). Four (0.3%) samples for amodiaquine and 37 (2.5%) samples for desethylamodiaquine were excluded from the analysis because of unreliable information on the time of sample collection and/or because the findings for those samples were deemed biologically implausible after inspection of the PK profile. In the final analysis, 1,456 (99.7%) samples for amodiaquine and 1,423 (97.5%) samples for desethylamodiaquine were included. Out of these, the concentrations in 803 (55.2%) samples for amodiaquine and 13 (0.9%) samples for desethylamodiaquine were below the LLOQ.

**TABLE 2:** Descriptions of population pharmacokinetic and noncompartmental studies^*a*^

Country	Study description (authors [reference(s)])	Treatment (protocol)	Study population	Formulation	Manufacturer	No. of patients	Sampling schedule (protocol)	Sample collection	Sample storage and assay	No. of samples per patient^*b*^	LLOQ of AQ/DEAQ concn (ng/ml)
Thailand^*c*^	Effect of pregnancy on PK and PD of amodiaquine and desethylamodiaquine (Tarning and colleagues [21, 24])	AQ (10 mg/kg) daily for 3 days, 200 mg amodiaquine hydrochloride (153 mg amodiaquine base)	Pregnant women (ages, 16 to 39 yr) in their 2nd and 3rd trimester (with follow-up after delivery)	AQ alone	Sanofi-Aventis, France	26 (7)^*d*^	0, 4, 24, 28, 48, 48.5, 49, 50, 51, 52, 54, 56, 58, and 72 h; 4, 5, 7, 14, 21, 28, 35, and 42 days	A sample was drawn from a catheter during the first 3 days and thereafter by venous puncture and placed into lithium heparin tubes	Samples were stored at −20°C and analyzed by LC-MS/MS	14/22 (14/22)	1/2
Kenya^*c*^	Efficacy of fixed vs nonfixed dose of ASAQ (Jullien et al. [23])	Two tablets of AS-AQ at a fixed dose (100/270 mg) or 4 tablets of AS (50 mg) + 4 tablets of AQ (153 mg) daily for 3 days, 353/200 mg amodiaquine hydrochloride (153/270 mg amodiaquine base)	Adults (ages, 18 to 60 yr)	AS + AQ at a fixed dose and as a loose formulation	Sanofi-Aventis, France	53	Before 1st dose, 15 min to 4 h after 1st dose, 15 min to 4 h after 2nd dose, just before 3rd dose, 15 min to 4 h after 3rd dose, days 7, 14, 21, and 28		Samples were analyzed by HPLC	4/8	1/1
Uganda^*e*^	Determine PK parameters for AS and AQ in children (Mwesigwa et al. [25])	AS (50-mg tablets at 4 mg/kg twice a day for 3 days) + AQ (200-mg tablets at 10 mg/kg once a day on the first 2 days and 5 mg/kg on the third day), 200 mg amodiaquine hydrochloride (153 mg amodiaquine base)	Children (ages, 5 to 13 yr)	AS + AQ, loose formulation	Sanofi-Aventis, France	20	Just prior to 3rd dose and at 2, 4, 8, 24, and 120 h after 3rd dose	A venous sample was drawn into potassium oxalate-sodium fluoride tubes	Samples were stored at −80°C and analyzed by LC-MS/MS	2/6	5/5
Burkina Faso^*c*^	Compare bioavailability of fixed doses of AS and AQ vs AS and AQ separately (Stepniewska et al. [22])	AS-AQ at a fixed dose (one dose of 25/67.5 mg/kg for children <12 mo of age or two doses for children ages 12 to 60 mo) or AS (50-mg tablet, a half tablet for children <12 mo of age and one tablet for children ages 12 to 60 mo) + AQ (153 mg, a half tablet for children <12 mo of age and one tablet for children ages 12 to 60 mo) daily for 3 days, 200 mg amodiaquine hydrochloride (153 mg amodiaquine base)	Children (ages, 1 to 5 yr)	AS + AQ at a fixed dose and as a loose formulation	Sanofi-Aventis, France	61	Before the 1st dose, 4 h after the 3rd dose, and then at days 7 and 14 and days 21 and 28	A venous sample was collected in lithium heparin tubes	Samples were stored at −20°C and analyzed by LC-MS/MS	2/3	1/1
Ghana^*c*^	Compare the effect of AS on AQ (comparison between AQ and loose formulations of AS and AQ) (Adjei et al. [20])	AQ (10-mg/kg single dose) or AS (4-mg/kg single dose) + AQ (10-mg/kg single dose) daily for 3 days, 200 mg amodiaquine hydrochloride (153 mg amodiaquine base)	Children (ages, 1 to 14 yr)	AS + AQ, loose formulation	Pfizer, Dakar, Senegal	101	Before the dose on days 3 and 7	A venous sample was collected into heparinized polypropylene tubes	Samples were stored at −20°C and analyzed by HPLC	1/2	10/10

a All samples were venous plasma. AS, artesunate; AQ, amodiaquine; LLOQ, lower limit of quantification; DEAQ, desethylamodiaquine; LC-MS/MS, liquid chromatography-tandem mass spectrometry; HPLC, reverse-phase high-performance liquid chromatography.

b Median number of amodiaquine/desethylamodiaquine samples per subject; values in parentheses are for the same women at 3 months postdelivery.

c Population PK study.

d The same women were sampled again at 3 months postdelivery during another malaria episode.

e Noncompartmental pharmacokinetic analysis.

### Population pharmacokinetic model.

**FIG 1:**
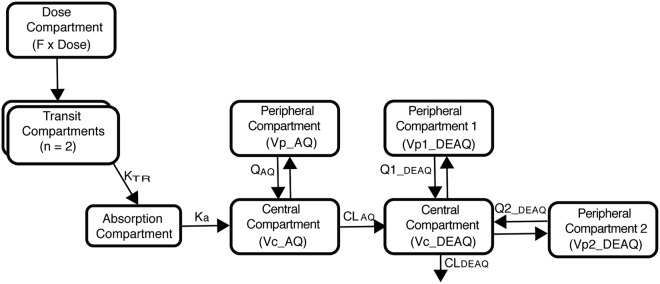
Structure of the PK model of amodiaquine and desethylamodiaquine. Abbreviations: *F*, oral bioavailability; *K*_TR_, first-order transit rate constant; *K_a_*, absorption rate constant; AQ, amodiaquine; DEAQ, desethylamodiaquine; CL, clearance; *Vc*, central volume of distribution; *Q*, *Q*_1_, and *Q*_2_, intercompartmental clearances; *Vp*, *Vp*_1_, and *Vp*_2_, peripheral volumes of distribution.

The observed amodiaquine data were best described by a two-compartment disposition model, and two transit compartments successfully described the absorption phase, while desethylamodiaquine was best described by three-compartment disposition kinetics. Replacement of the structural model with a semimechanistic hepatic model did not result in a better fit and was not pursued further. The use of the M3 method to handle LLOQ values did not significantly affect the population parameter estimates, but the model estimation process was unstable and required much longer run times than the M6 method, which was therefore used throughout the modeling process. The final structural model is depicted in Fig. 1, and the parameter estimates, together with their precision obtained from a nonparametric bootstrap, are reported in Table 3.

**TABLE 3:** Parameter estimates of the population pharmacokinetic model for amodiaquine and desethylamodiaquine^*f*^

Drug and parameter	Typical value^*a*^		BSV or BOV^*a*^^,^^*b*^				
	Value (% RSE)	95% CI	BSV (% RSE)	BOV (% RSE)	% shrinkage		95% CI
					Eta	Epsilon	
Amodiaquine							
*K_a_* (1/h)	0.589 (23)	0.409, 0.905		78.5 (12)	41.7		62.0, 96.5
MTT (h)	0.236 (26)	0.161, 0.334		93.4 (16)	61.8		60.6, 120
NN	2.00 (78)	1.09, 6.31					
*F*	1 Fixed			30.9 (8.0)	27.1		26.5, 36.0
CL_AQ_^*c*^ (liters/h)	2,960 (4.4)	2,600, 3,118	32.2 (13)		44.3		24.2, 39.5
*Vc*_AQ_^*c*^ (liters)	13,500 (19)	7,423, 17,824	53.1 (30)		55.4		19.4, 79.5
*Q*_AQ_^*c*^ (liters/h)	2,310 (9.2)	1,877, 2,722					
*Vp*_AQ_^*c*^ (liters)	22,700 (10)	17,956, 27,114					
Additive error^*d*^ (ng/ml)	LLOQ/5 + 0.445 (19)	LLOQ/5 + 0.249, 0.609				26.7	
Proportional error (%)	19.9 (11)	16.1, 24.6					
Desethylamodiaquine							
CL_DEAQ_^*c*^ (liters/h)	32.6 (2.9)	29.7, 33.4	20.0 (10)		31.3		15.5, 23.5
*Vc*_DEAQ_^*c*^ (liters)	258 (12)	201, 318	67.2 (21)		59.3		36.0, 89.2
*Q*_1DEAQ_^*c*^ (liters/h)	154 (6.6)	131, 171					
*Vp*_1DEAQ_^*c*^ (liters)	2,460 (5.9)	2,129, 2,677					
*Q*_2DEAQ_^*c*^ (liters/h)	31.3 (6.2)	26.8, 34.3					
*Vp*_2DEAQ_^*c*^ (liters)	5,580 (4.3)	4,968, 5,904					
Additive error^*d*^ (ng/ml)	LLOQ/5 Fixed					16.5	
Proportional error (%)	24.2 (4.0)	22.2, 25.9					
Covariate effects							
PMA_50_ for AQ^*e*^ (time [mo] from conception)	11.8 (4.6)	10.50, 12.70					
Hill factor for AQ^*e*^	3.6 (4.0)	3.23, 3.80					
PMA_50_ for DEAQ^*e*^ (time [mo] from conception)	12.9 (5.7)	11.60, 14.30					
Hill factor for DEAQ^*e*^	3.22 (4.7)	2.85, 3.43					
Effect of first dose on *F* (%)	−22.4 (19)	−32.0, −15.6					

a The precision of the parameter estimates was assessed using a nonparametric bootstrap of the final model (*n* = 500). The relative standard errors were calculated as 100 × (standard deviation/mean), while the confidence intervals were obtained on the basis of the empirical percentiles of the bootstrap estimates.

b Between-subject and between-occasion variability were assumed to be log-normally distributed and are reported as the approximate percent coefficient of variation.

c All clearances and volumes of distribution refer to a patient weighing 50 kg, the median weight in the data set.

d For the data contributed by each study, the additive error was fixed to 20% of the lower limit of quantification (LLOQ) in that study plus an estimated parameter. For desethylamodiaquine, this extra parameter was not significantly different from zero, so the additive error was fixed to the lower bound of LLOQ/5.

e Estimated using prior functionality in NONMEM.

f Abbreviations: AQ, amodiaquine; DEAQ, desethylamodiaquine; BSV, between-subject variability; BOV, between-occasion variability; RSE, relative standard error; *K_a_*, absorption rate constant; MTT, mean transit time; NN, number of transit compartments; *F*, relative bioavailability; CL, clearance; *Vc*, central volume of distribution; *Q*, *Q*_1_, and *Q*_2_, intercompartmental clearances; *Vp*, *Vp*_1_, and *Vp*_2_, peripheral volumes of distribution; PMA_50_, time to reach 50% of clearance maturation; Hill factor, steepness of the clearance maturation curve.

**FIG 2:**
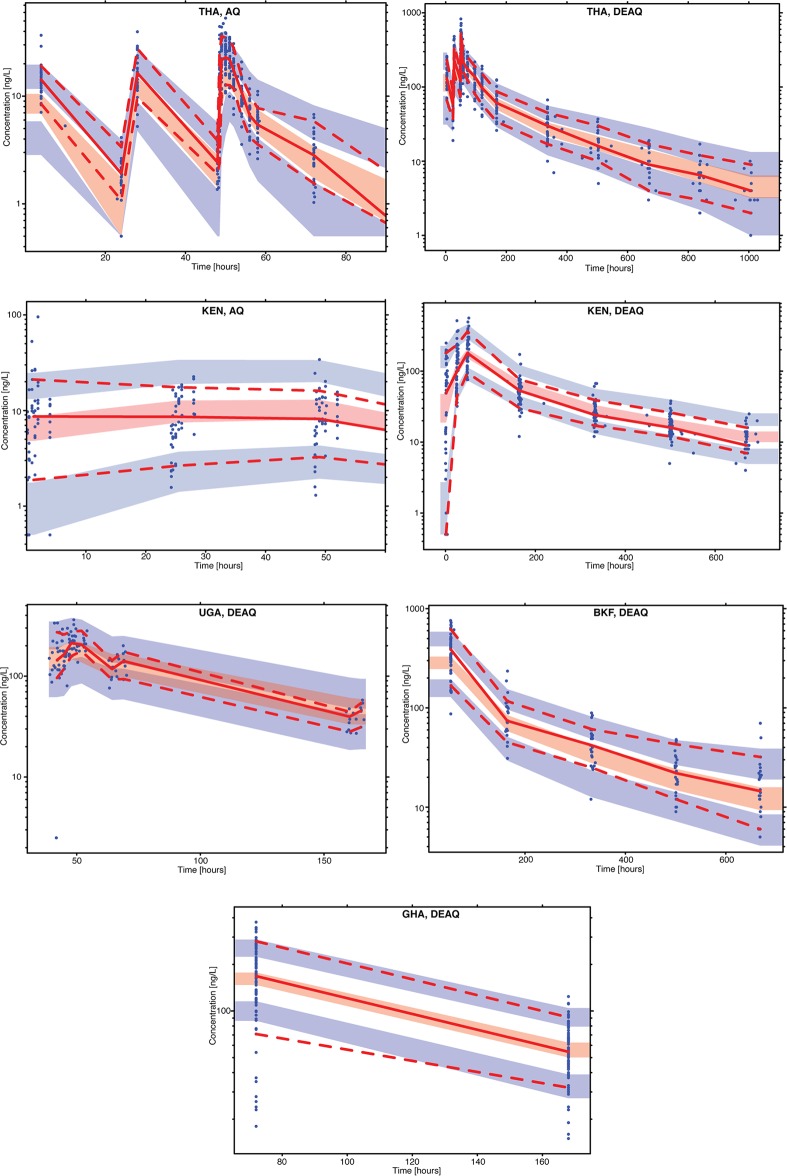
Visual predictive check of the final model describing the plasma concentrations of amodiaquine (AQ) and desethylamodiaquine (DEAQ) versus time in uncomplicated malaria patients from Thailand (THA), Kenya (KEN), Uganda (UGA), Burkina Faso (BKF), and Ghana (GHA). Open circles are the observed data points; solid and dashed lines are the 50th, 5th, and 95th percentiles of the observed data; shaded areas are the simulated (*n* = 1,000) 95% confidence interval for the same percentile. The *y* axis represents the plasma concentration on the log scale. Censored data points below the lower limit of quantification were imputed as LLOQ/2 and included in the calculation of percentiles for the observed and the simulation data. The VPC for amodiaquine for both Thailand and Kenya was cut at times of 90 and 60 h, respectively, since beyond these times the concentrations from both observed and simulated data were below the LLOQ.

The model adequately described all observed data from the included studies, as shown by the study-stratified visual predictive check (VPC) plots in Fig. 2. VPCs stratified by age and basic goodness-of-fit diagnostic plots are presented in Fig. S1 and S2 in the supplemental material, respectively. Even though some small trends can be observed for amodiaquine for the first hours after the dose in some of the contributed studies, these plots showed no overall obvious model misspecification and suggested that the developed model has adequate predictive performance, especially for desethylamodiaquine, which is the main driver of the therapeutic outcome.

### Covariate effect.

**FIG 3:**
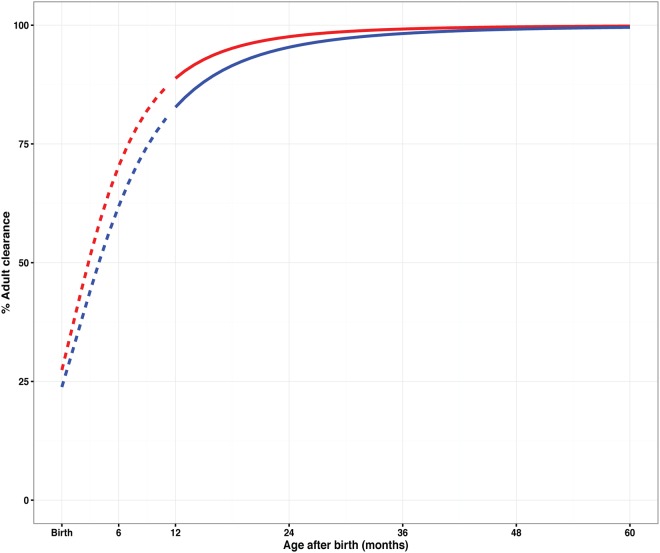
Clearance maturation for amodiaquine (red line) and desethylamodiaquine (blue line) expressed as a fraction of adult clearance predicted from the PK model plotted against postnatal age (assuming that birth occurred at term). The dashed lines indicate the section of the maturation curve that falls in the age range below that observed in the study data and are therefore based on an extrapolation.

Allometric scaling with body weight on all clearance and volume parameters improved the model fit substantially (change in the objective function value [ΔOFV] = −446). Inclusion of a maturation function for the clearance of desethylamodiaquine improved the model further (ΔOFV = −34.6; 2 degrees of freedom [df]; *P* < 0.001) and decreased the between-subject variability (BSV) of desethylamodiaquine clearance from 24% to 19.1%. A similar maturation effect did not reach statistical significance for amodiaquine (ΔOFV = −3.1; 2 df; *P* = 0.21) and decreased the BSV on clearance only slightly, from 62.6% to 61.7%, but it was retained in the final model for consistency between the parent compound and the metabolite. The final parameter estimates of the maturation function were close to the prior values used to stabilize the model; clearance was found to reach 50% of weight-adjusted adult values at 2.8 months of age (95% confidence interval [CI], 1.5 to 3.7 months of age) and 3.9 months of age (95% CI, 2.6 to 5.3 months of age) (assuming a standard 9-month gestation) for amodiaquine and desethylamodiaquine, respectively. The maturation effect is displayed in Fig. 3, which shows that 95% of the adult value is reached by 2 years of age. Finally, the model estimated the bioavailability of the first dose in the treatment regimen to be 22.4% (95% CI, 15.6 to 31.9%) lower than that on the second and third days of treatment (ΔOFV = −18.9; 1 degree of freedom; *P* < 0.001; decrease in the between-occasion variability [BOV] on bioavailability, 33% to 31%). Inclusion of the drug formulation and hemoglobin (HB) concentration on bioavailability did not improve the model fit, nor did the inclusion of the hemoglobin concentration on amodiaquine or desethylamodiaquine clearance.

### Variability.

Even after adjusting for all covariate effects described above, a large amount of BSV and BOV was still identified. A large BOV in absorption parameters (>78%), a large BSV in the central volume of distribution for both amodiaquine and desethylamodiaquine (>50%), a moderate BSV in clearance for both amodiaquine and desethylamodiaquine (20 to 33%), and a moderate BOV for bioavailability (30.9%) were observed. Moderate to high eta shrinkages (30 to 60%) were observed for parameter estimates, while epsilon shrinkages were low (<30%), as summarized in Table 3.

### Simulations.

**FIG 4:**
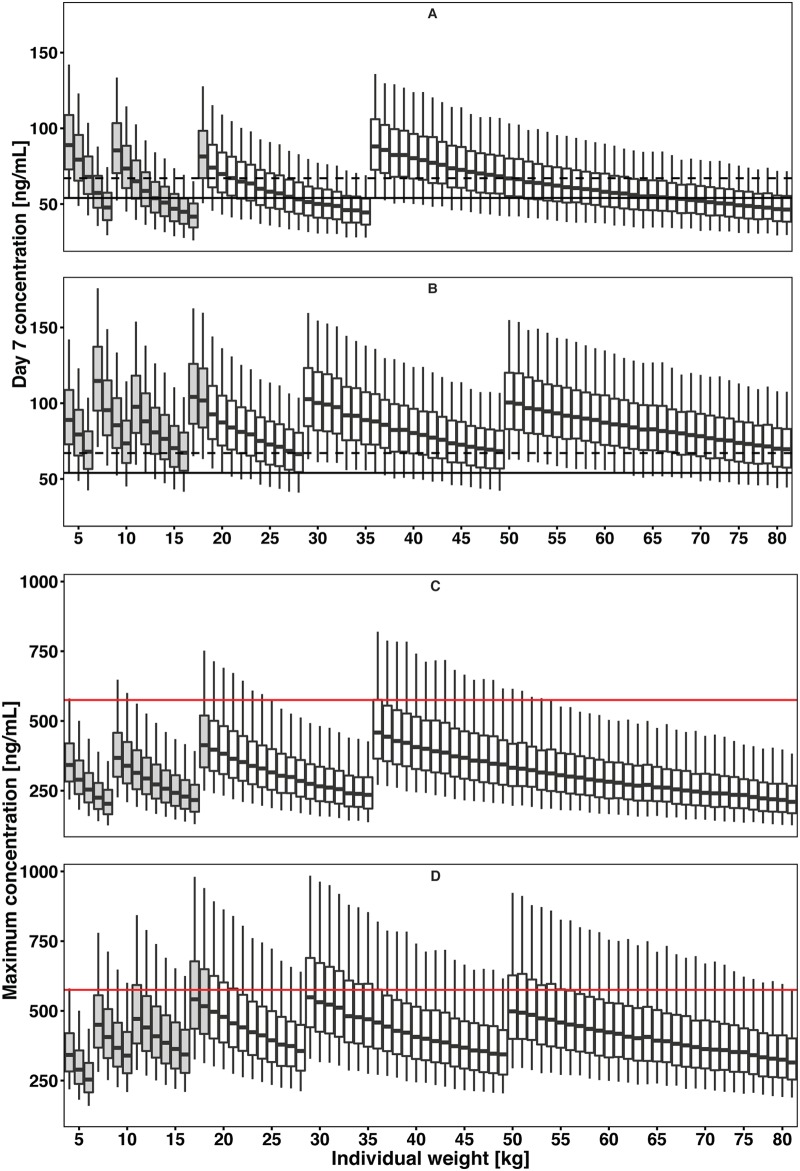
Simulation results of current recommended and optimized dose regimens for amodiaquine. (A and C) Day 7 plasma desethylamodiaquine concentration (A) and maximum concentration of desethylamodiaquine (C) based on the current recommended dose regimen. (B and D) Predicted desethylamodiaquine day 7 concentration for the optimized dose regimen designed to achieve a concentration ≥75% above the threshold value (B) and *C*_max_ of desethylamodiaquine for the optimized dose regimen (D). The black dashed and solid lines in panels A and B are the median and 80% value of median of the simulated day 7 plasma desethylamodiaquine concentration for the typical patient (representing the expected exposure level from the current dosing recommendation), respectively, and the red lines in panels C and D represents the *C*_max_ upper threshold (575 ng/ml). Simulations for each weight are presented as a box plot for the median and 25th and 75th percentiles, with whiskers representing the 5th and 95th percentiles. The boxes in gray indicate that the simulation for that age range is based on an extrapolation, since no PK data for children of that age were available.

The final model was used to evaluate the exposure achieved with the current dosing recommendations (26), and the results are summarized in Fig. 4A. Children who weigh 8 kg, 15 to 17 kg, or 33 to 35 kg and patients who weigh >62 kg achieve desethylamodiaquine day 7 concentrations that are, on average, 25% lower than the concentration for a 50-kg patient. Population-based simulations using the final PK model suggested that higher dosages are needed to achieve equivalent exposures in all body weight groups, raising the dosing recommendations for amodiaquine from 7.5 to 16.9 mg/kg/day to 9.8 to 19.9 mg/kg/day in certain patient groups (Fig. 4; Table 4). It is important to note that Fig. 4 shows simulated patients, including some with ages below the age range of the observed data.

**TABLE 4:** Current dose regimen and optimized dose regimen based on simulations for amodiaquine

Current manufacturer dose regimen			Proposed dose regimen		
Body wt (kg)	No. of tablets/day, tablet strength	Total amodiaquine dose (mg)	Body wt (kg)	No. of tablets/day, tablet strength (mg)	Total amodiaquine dose (mg)
4 to 8	1, 67.5	67.5	4 to 6^*a*^	1, 67.5	67.5
9 to 17	1, 135	135	7 to 10^*b*^	1, 135	135
18 to 35	1, 270	270	11 to 16	1.5, 135^*c*^	202.5
≥36	2, 270	540	17 to 28	2.5, 135^*c*^	337.5
			29 to 49	2, 270	540
			≥50	3, 270	810

a A substantial number of simulated patients (78%) in this weight band were younger (<1 year) than those available in the data set.

b A proportion of the simulated patients (38%) in this weight band were younger (<1 year) than those available in the data set.

c In these weight bands, as an alternative to splitting tablets, one could suggest using tablets of different strengths. Either option is viable, according to the preference of the caregiver.

The predicted lower target concentration for efficacy, i.e., 80% of the median desethylamodiaquine concentration at day 7 in a 50-kg patient taking 540 mg daily for 3 days (15 mg/kg/day), was 54.0 ng/ml. The proposed optimized dosing regimen (Table 4) resulted in equivalent exposures across weight bands (Fig. 4B). In particular, higher milligram-per-kilogram doses were needed for small children, consistent with the nonlinear effect of body size on clearance, described by allometric scaling.

## DISCUSSION

This is the first pooled population PK analysis of amodiaquine and desethylamodiaquine. The analysis used individual patient data from six studies (five cohorts) of the antimalarial therapy amodiaquine, given alone or in combination with artesunate (20–25), covering patient populations with a large range of characteristics (in terms of weight, age, and ethnicity) and covering a large range of treatments and study protocols. Consistent with other studies, the final PK model included a two-compartment model for the amodiaquine concentration-time profile and a three-compartment model for desethylamodiaquine. In addition to including the effect of body size on the clearance of both compounds with allometric scaling, the maturation profile of the clearance of both compounds was included in the model to describe the kinetics in children. To ensure exposure equivalent to that in adults, and hence assuming that this would lead to improved efficacy and curative concentrations, simulations with the final model of desethylamodiaquine indicated that higher doses of amodiaquine may be needed for some weight ranges (8 kg, 15 to 17 kg, 33 to 35 kg, and >62 kg), but this finding needs further validation.

The effect of the drug formulation or coadministration with artesunate on bioavailability was not significant in this analysis. Previously, efficacy was found to be higher for amodiaquine when given as a fixed-dose formulation with artesunate (6), suggesting that a fixed-dose formulation may directly increase bioavailability or improve adherence, leading to higher exposure, as measured by the area under the plasma concentration-time curve (AUC). Only a few patients included in the analysis received amodiaquine alone; this group included all of the patients from the Thailand study and only 6% (15/256) of the patients from studies conducted in Africa. Moreover, the patients treated with amodiaquine alone received a higher dose on a milligram-per-kilogram basis than those that received the loose formulations or the fixed-dose combination therapy. These two factors possibly limited the power of the analysis to explore formulation effects. Similarly, all pregnant patients and those infected with Plasmodium vivax were from one study in Asia; thus, there was little power to separate study-specific effects from any pregnancy, regional, or Plasmodium species effect on the PK parameters.

The structural PK model identified here matches that proposed by Tarning et al. (21) in pregnant women in Thailand. This is not surprising, since their study contributed the most intensively sampled PK profiles included in this pooled analysis. Parameter estimates were similar between the two analyses, but the central volume of distribution was larger in this pooled analysis, and, as expected, pooling of data from several studies resulted in a larger BSV (21). This could be due to differences in the body size and composition between the Asian and African populations, or it could simply be a consequence of different study sample sizes. Unfortunately, patient height data were unavailable from the African studies, and other body size descriptors (i.e., fat-free mass or body mass index) could not be explored as an alternative to total body weight.

Amodiaquine bioavailability was found to be 22.4% lower on the first day of treatment than on the second and third days, possibly due to an increased absorption of amodiaquine as a result of treatment and a general improvement in malaria disease status. Winstanley et al. (27) reported lower AUCs for amodiaquine in malaria patients than in healthy volunteers, and this may be partly explained by the lower bioavailability due to a disease effect that is more pronounced on the first day than during convalescence. Similarly, a nearly 2-fold (1.72-fold) relative increase in bioavailability has been observed in studies of mefloquine, where administration is delayed until 48 hours after the first dose of artesunate (28). A similar increase in oral bioavailability during treatment has been demonstrated in malaria patients receiving dihydroartemisinin-piperaquine and for naphthoquine when given in combination with artemisinin in pediatric malaria patients (29–32). In contrast, a higher bioavailability was estimated for artesunate during the acute malaria phase than during the convalescent phase in a recent study (33). Taken together, there might be an emerging trend of lower bioavailability during acute phases of malaria illness for the longer-acting compound in ACT treatments, warranting further investigation. A similar parallel has been noted with antibiotic use in patients with sepsis (34).

The disease state may also alter absorption parameters (35), but the current analysis could not assess the effect of acute illnesses on absorption, as it included only malaria patients and not healthy individuals. There was considerable heterogeneity in malaria symptoms and parasitemia, but attempts to include an effect of various parasite densities in the model were unsuccessful, possibly because it was confounded by other factors. The principal plasma protein binding site for amodiaquine is in α_1_-acid glycoprotein (13), which is upregulated during malaria (36, 37), resulting in increased binding of the drug to this protein. The variation in α_1_-acid glycoprotein levels could also explain part of the high between-subject variability in the apparent volume of distribution found in our analysis.

The large estimates of between-occasion variability could be a result of sparse data, and some of the studies (23, 25) included in this analysis did not measure drug concentrations on the first and second days of treatment and/or provided only approximate information about the timing of sample collection (i.e., only the day and not the exact time was available). Despite this, the effect of lower oral bioavailability for the first dose was confirmed even in a separate analysis executed only on data from studies that included sufficient information.

PK parameter estimates from adults, adjusted for the effects of body weight and size by allometric scaling, often provide poor predictions of drug clearance in young children (38), especially for those less than 1 year of age. This is because PK can change rapidly with postmenstrual age (PMA) (39–41) in the first years of life. Age maturation of clearance rates has been reported for various pediatric drugs (16, 42–45). In addition, the clearance of amodiaquine depends on the enzyme CYP2C8, which is closely related to CYP2C9 (46), whose isozymes are known to exhibit maturation effects (38). Similar to the approach used in previous studies of other drugs (42, 44), a sigmoidal maximum-effect function was used to model the maturation of clearance as a function of PMA. No previous estimates have been presented for amodiaquine or desethylamodiaquine, but the estimates of the PMA at which 50% enzyme maturation is achieved (PMA_50_) presented here result in half-maximum maturation of amodiaquine and desethylamodiaquine clearance at 2.8 and 3.9 months after birth, respectively, for babies born at term. This is in line with the range reported for other drugs (0.7 to 12 months after birth, respectively, for babies born at term), as summarized by Holford et al. (47). The estimates of the Hill coefficients were also consistent with those reported in previous studies (2 to 4), indicating a relatively sharp maturation curve plateauing at about 2 years after birth. Amodiaquine and desethylamodiaquine clearance reached at least 95% of the adult values at approximately 21 months after birth, suggesting that body size is the main pharmacokinetic determinant for children older than 2 years of age. Unfortunately, the present analysis did not include data from children younger than 1 year of age, most likely because children <1 year of age are commonly administered artemether-lumefantrine or quinine; thus, few studies have investigated the PK of artesunate-amodiaquine in infants <1 year of age. Due to the lack of available data for young children, the parameter values of the maturation function are influenced by information published for other drugs with CYP-dependent clearance. Other significant effects not included in our model may be relevant for very young children, including limited absorption capabilities.

Appropriate drug dosing in children is particularly challenging (48), and a number of studies have reported suboptimal dosing of children receiving antimalarial treatment (14–19). Simulations presented in this analysis suggest that optimal plasma concentrations require doses of amodiaquine higher than those currently recommended for children weighing 8 kg, 15 to 17 kg, or 33 to 35 kg (Fig. 4). The same is true for patients weighing >62 kg. The proposed alternative dosing guidelines were developed on the basis of the strengths of currently available amodiaquine tablets, which are manufactured in strengths of 67.5 mg and multiples thereof. The amodiaquine dose adjustment proposed would increase complexity due to additional weight bands and would need to be implemented with suitable additional training and tools (Table 4). However, before routine implementation, the suggested regimen should be evaluated in prospective trials for efficacy and safety. Since amodiaquine and artesunate are often coformulated in a fixed-dose combination, the proposed dose changes would also increase the artesunate dose from 2.8 to 6.3 mg/kg/day to 3.6 to 7.4 mg/kg/day, which remains within the 2- to 10-mg/kg/day range recommended by WHO (49).

Simulations were used to propose optimized doses for the wide range of body weights recommended by the manufacturers for artesunate-amodiaquine administration (26). The simulated patients included very young children 2.5 to 15.2 months old with weights of 4.5 kg to <6.0 kg, which are less than those of any of the patients for which drug concentration-time data were available. For those patients, the proposed optimized dosages are based on the inclusion of allometric scaling and, even more critically, the maturation effect in very young children. Including these young children in the simulations could provide some understanding of the expected exposure in this group. Thus, the estimated exposure values and the suggested dose optimization in this subgroup of the patients should be treated as extrapolations and interpreted with caution. There is an urgent need to further investigate individuals in this age range.

The proposed new dosing recommendation suggests a substantial dose increase for particular patient groups compared to the currently recommended values; e.g., the dose for patients weighing 17 kg and 29 kg would change from 135 mg to 337.5 mg and 270 mg to 540 mg, respectively. As a consequence of the dose increase, these patients will experience higher peak concentrations, but the predicted median values of 560 ng/ml and 552 ng/ml for patients with weights of 17 kg and 29 kg, respectively, are within the range of peak concentrations found in other clinical trials of amodiaquine (49).

This analysis assumes that the concentration threshold for efficacy in the reference patient of 50 kg is equally applicable to younger individuals. However, the exposures required for efficacy in children may be higher than those required for efficacy in adults. This is because children tend to have lower levels of partial immunity in areas where malaria is endemic (50) and, consequently, higher parasite counts, lower hemoglobin levels, and a higher risk of treatment failure and progression to severe malaria. Yet, data on the safety of amodiaquine and any possible age dependence are limited. However, it will be necessary to collect safety data for this new dose before implementation. Further studies of the proposed dosing schedule are thus needed to confirm safety and efficacy, especially in very young children.

As with all PK studies, there are limitations to the present analysis. Data for infants younger than 1 year of age were not available, thus resulting in uncertainty in the estimated maturation of clearance. Similarly, children younger than 12 years of age contributed a median of two samples per patient. The concentrations of amodiaquine were mostly below the LLOQ, making it difficult to characterize the pharmacokinetics of the parent compound in this age group. On the other hand, desethylamodiaquine concentrations were generally detectable, and thus, it was possible to obtain, even in this age group, a reasonably accurate description of the pharmacokinetics of the metabolite, which is the main source of pharmacological action. The description of pharmacokinetics in children was compensated for by including existing information on the maturation of the enzymes involved in metabolizing amodiaquine. This step made the estimates coherent with the general maturation profile (i.e., the enzymes are mature before 2 to 3 years of age), and it resulted in narrow bootstrap confidence intervals for the maturation parameters. Still, the results need confirmation. Precise information on the time of dose and/or the sampling time was not always available in some studies; hence, it was assumed that the times were consistent with the protocol schedule. In addition, different assays with different limits of quantification were used across the studies included in this analysis.

In conclusion, pooled individual concentration-time data for amodiaquine and its metabolite (desethylamodiaquine) were described using population PK modeling. Amodiaquine was described accurately by a two-compartment disposition model followed by a three-compartment disposition model for desethylamodiaquine. This study is the first to model the maturation of amodiaquine and desethylamodiaquine clearance as a function of postmenstrual age and, hence, provides the basis for further analysis of whether infants and young children achieve exposures equivalent to those in adults. The differences in amodiaquine PK between adults and children can largely be accounted for by body weight and the maturation effect. Amodiaquine exposures after standard daily oral doses were lower in small children and in patients weighing more than 62 kg. The body weight-adjusted dosing regimen proposed in this study is expected to achieve similar exposure levels in all patients without an increased risk of acute toxicity.

## MATERIALS AND METHODS

### Clinical studies and data.

This pooled analysis used data from previous studies conducted in different populations and across different geographical locations. Clinical and PK data from six studies (five cohorts) conducted in five countries (Burkina Faso, Ghana, Kenya, Uganda, and Thailand) were shared with the WorldWide Antimalarial Resistance Network (WWARN) and used for this analysis (Table 2). Relevant studies were identified by searching PubMed, Embase, Google Scholar, ClinicalTrials.gov, and conference proceedings, using the key words “amodiaquine pharmacokinetics” or “amodiaquine concentrations” and “clinical study.” The first and last authors of relevant studies were contacted and invited to join this pooled analysis. Participating authors agreed to the WWARN terms of submission, which ensures that all data uploaded are anonymized and have been obtained with informed consent and in accordance with any laws and ethical approvals applicable in the country of origin (51). Information provided by each study included symptoms, dosing, drug concentrations, parasitemia, and clinical and laboratory data over time. Patients were administered either amodiaquine monotherapy or artesunate plus amodiaquine as a loose formulation or fixed-dose combination therapy. Treatment was given once daily for 3 days at a target dose of 10 mg/kg of body weight (Table 2). Data from 261 patients were pooled. Patients reflected a wide range of ages (1 to 60 years) and weights (6.5 to 93 kg). Only 26 pregnant women were included in the data pool. Detailed information on recruitment of study participants, randomization, and follow-up can be found in the reports of the respective studies (20–25). Information on sample collection, storage, and the assays used is summarized in Table 2. Individuals were excluded from the analysis if information on drug dosage was unavailable, while protocol times were imputed for patients with missing information on the exact dosing times. All administered doses were converted to the amount of amodiaquine base (in milligrams) before modeling. The data sets for each study were merged and formatted for subsequent analysis using Stata software (version 11; StataCorp, College Station, TX, USA) according to the Clinical Pharmacology Data Management and Statistical Analysis Plan (52, 53).

### Structural model.

Pharmacokinetic compartmental models were fitted to the observed concentration-time data for amodiaquine and desethylamodiaquine using nonlinear mixed-effects modeling with NONMEM software (version 7.3; Icon Development Solutions, Ellicott City, MD). Amodiaquine and desethylamodiaquine concentration measurements were fitted using the first-order conditional estimation method (54) with the eta-epsilon interaction. The Perl-speaks NONMEM, Xpose (version 4.3.5), Pirana (55), and R (version 3.1.2) (56) programs were used for automation and diagnostics during the model-building process. Nested models were assessed by their objective function value (OFV), computed by NONMEM to be proportional to −2 times the log likelihood of the data. A decrease in the OFV of at least 3.84 points was considered a statistically significant difference with *P* equal to 0.05, when comparing two hierarchical models with one parameter difference (χ^2^ distribution with 1 degree of freedom [df]).

The structural (base) model was established using the most densely sampled clinical trial from the pooled data. Subsequently, data from different studies were added one by one in order of data richness, and each time the model was fit to the new data, reassessed, and modified if necessary (57).

One-, two-, or three-compartment disposition models with first-order elimination were investigated for amodiaquine. First-order absorption models with and without lag time and transit compartment absorption (58) were tested to describe drug absorption. Thereafter, the parameters for the best-performing amodiaquine model were fixed to the final estimates, and the structural disposition model for desethylamodiaquine was investigated. Amodiaquine was assumed to be completely and irreversibly metabolized to desethylamodiaquine (7), and a molar conversion factor was included to account for the difference in molecular weight between the two compounds. One-, two-, and three-compartment disposition models with first-order elimination were evaluated for desethylamodiaquine. Finally, the amodiaquine and desethylamodiaquine concentrations were fitted simultaneously and the model was reevaluated. Additionally, we tested a semiphysiological model with hepatic extraction, implemented as previously described (59), with the aim of describing both hepatic clearance and first-pass extraction using the single parameter of hepatic intrinsic clearance (12, 60).

Several approaches to handling values below the lower limit of quantification (LLOQ) were tested (61, 62), ignoring the data below the LLOQ (M1), imputing LLOQ/2 for the first value in a consecutive series followed by ignoring the subsequent data below LLOQ (M6), or applying a likelihood-based approach (M3). Model stability and the robustness of the parameter estimates, as well as model run times, were considered when deciding on the approach for handling data below the LLOQ. All data below the LLOQ were retained in simulation-based diagnostics, i.e., visual predictive checks (VPCs).

### Effect of body size and age.

The impact of body weight was evaluated by allometric scaling of all clearance (exponent of 0.75) and volume (exponent of 1) parameters for amodiaquine and desethylamodiaquine, considering the strong biological prior of this relationship (47). A maturation function was investigated to characterize the age-related changes in clearance (39, 63). The individually predicted value of clearance was obtained by combining both the effect of size (allometric scaling) and the developmental process (maturation function), as shown in Equation 1:

(1) $$\text{CL}={\text{CL}}_{\text{TV}}\cdot (\frac{\text{BW}}{{\text{BW}}_{\text{TV}}}{)}^{3/4}\cdot \frac{{\text{PMA}}^{\text{Hill}}}{{\text{PMA}}^{\text{Hill}}+{\text{PMA}}_{50}^{\text{Hill}}}$$

where PMA is postmenstrual age (gestational age plus postnatal age), PMA_50_ is the PMA at which 50% enzyme maturation is achieved, and Hill is a shape factor for the relationship. CL is clearance, CL_TV_ is the clearance for a typical patient, BW is the individual body weight, and BW_TV_ is the body weight of a typical patient.

The reason for using PMA for the maturation function is that maturation begins *in utero* (39) and at birth organs have already achieved a certain level of maturation. No information on the duration of gestation for the individual children was available, so PMA was obtained by simply adding 9 months to the postnatal age, assuming no premature births. For ease of interpretation, the results are presented and discussed in terms of postnatal age for a baby born at term. As no data were available for children younger than 1 year of age (among the studies included), prior information was used to stabilize the parameters of the maturation function to physiologically plausible values, comparable to those used for other drugs (16, 40, 42–44, 64). The Prior functionality in NONMEM was employed for this (65), assuming weakly informative priors (10% uncertainty) for the maturation curve and using values of 12 months for PMA_50_ and 3.5 for the Hill coefficient. These values are in line with the maturation profile of CYP450 enzymes (46) and generally in line with the maturation of most drug elimination pathways (47), so they were considered reasonable priors for amodiaquine, which is mainly metabolized by CYP2C8 (7–9).

### Stochastic model.

A log-normal distribution was assumed for between-subject variability (BSV) and between-occasion variability (BOV) in the PK parameters. The unexplained residual variability was modeled using a combined additive and proportional error model.

Drug concentration data from the different studies were obtained from different laboratories and with assays characterized by different limits of quantification, which might confer a different precision in the quantification of the lower concentrations in the pharmacokinetic profile. To account for this difference, we attempted to estimate a separate residual error for each study, but this proved unstable and resulted in the data sets with sparser sampling schedules being assigned unrealistically small errors and thus being overfit. As an alternative, we decided to conservatively add 20% of the LLOQ of each specific study to the estimated additive error component for the samples obtained in that study (Table 2). The value of 20% was chosen to remain consistent with the error level threshold generally used by analytical laboratories to define the limit of quantification when assays are developed according to drug development regulatory standards (66).

Whenever amodiaquine or desethylamodiaquine concentrations were detectable in the predose samples, those observed values were used to initialize all disposition compartments in the model to account for the prior presence of that drug in the body.

### Covariate analysis.

Predefined covariates considered for inclusion, besides weight and age (discussed above), were sex, drug combination (amodiaquine administered alone or in combination with artesunate) and formulation (loose formulation or fixed-dose combination), and the plasmodium species. A disease effect and any possible effects related to the patient's improving condition with treatment were investigated by testing the total parasite count and the hemoglobin (HB) concentration both at the baseline and on the days after initiating treatment and exploring whether the PK parameters varied after the initiation of treatment. Only three patients (1.2%) had missing parasitemia at the baseline; for these patients, the median value for their respective study population was used instead. Since some studies reported hematocrit (HT) and not hemoglobin (HB), the latter was estimated using equation 2 (67).

(2) $$\text{HB}=\frac{\text{HT}-5.62}{2.60}$$

The effect of covariates on PK parameters was assessed by using linear or exponential models for continuous covariates and additive proportional models for categorical covariates and by using a stepwise forward inclusion (*P* < 0.05) and backward elimination (*P* < 0.001) approach (68).

### Model assessment.

Model development was guided by improvements in the OFV, using the likelihood ratio test, and inspection of goodness-of-fit and other diagnostic plots. VPCs (69) stratified by country and age group were produced using Perl-speaks-NONMEM (PsN) with 1,000 simulations from the original data set. Eta and epsilon shrinkages were used to assess the reliability of empirical Bayes estimates and the power to detect model misspecifications in goodness-of-fit diagnostics (70). A nonparametric bootstrap with replacement (*n* = 500) was used to evaluate the robustness of the parameter estimates of the final model.

### Simulations.

Monte Carlo simulations were performed using the final PK model to explore the expected exposure and to evaluate optimal dosing regimens across different body weights. To ensure that the model would reflect exposure in accordance with the dosing guidelines for a specific population, in particular, underweight malaria parasite-infected children, *in silico* patients were generated by using individual demographic values (age and weight) from the studies included in the current analysis, plus other historical data (*n* = 748) from malaria patients (71–73) and routine clinical monitoring data for 1,580 children collected from the Bagamoyo Research and Training Center, a branch of the Ifakara Health Institute in Tanzania (unpublished). Pooling all these data, we obtained a database with 2,600 *in silico* patients covering a wide range of ages (2.5 months to 71.1 years) and body weights (4.5 to 93 kg). It is important to note that the age range of the patients used for simulations included ages lower than those for the children used to develop the final PK model.

Simulations using the final population PK model were repeated 5,000 times for each individual *in silico* patient. Subsequently, 3,000 exposures in each 1-kg weight band (1-kg intervals) were randomly drawn from the simulated results.

Desethylamodiaquine concentrations on day 7 were used as a proxy for efficacy, since they have been reported to correlate well with clinical cure rates (74). However, no target concentration value associated with clinical success is available in the published literature. The current dosing recommendation (49) results in reasonable cure rates in adults, suggesting that the exposure in a typical 50-kg adult with malaria is sufficient for treatment success. Thus, the simulated day 7 plasma concentration of desethylamodiaquine in this typical patient was assumed to be the relevant target concentration associated with clinical treatment success. The proposed dosing regimen was developed so that 75% of the patients in each 1-kg weight band were predicted to have day 7 desethylamodiaquine concentrations that were at least 80% of the target concentration. Also, the predicted maximum concentration (*C*_max_) of desethylamodiaquine was monitored to evaluate potential acute toxicity associated with increased dosing. The predicted 75th percentile of *C*_max_ in the patient weight group with the highest concentrations after receiving the currently recommend dosing was selected as a target level for potential acute toxicity. This reference target value was compared with the simulated exposures with the proposed dosing guidelines. Safety was assumed if the peak concentrations seen with the proposed guidelines did not significantly exceed the values already experienced by some patients with the current dosing recommendations.

## Supplementary Material

Supplemental file 1
