# Supplementary material for: Population Pharmacokinetics of the Antimalarial Amodiaquine: a Pooled Analysis To Optimize Dosing
Source: Antimicrob Agents Chemother. 2018 Sep 24;62(10):e02193-17. doi: 10.1128/AAC.02193-17 (PMC6153844; doi:10.1128/AAC.02193-17)
Supplement: Supplemental file 1 [file zac010187500s1.pdf]

Supplemental material

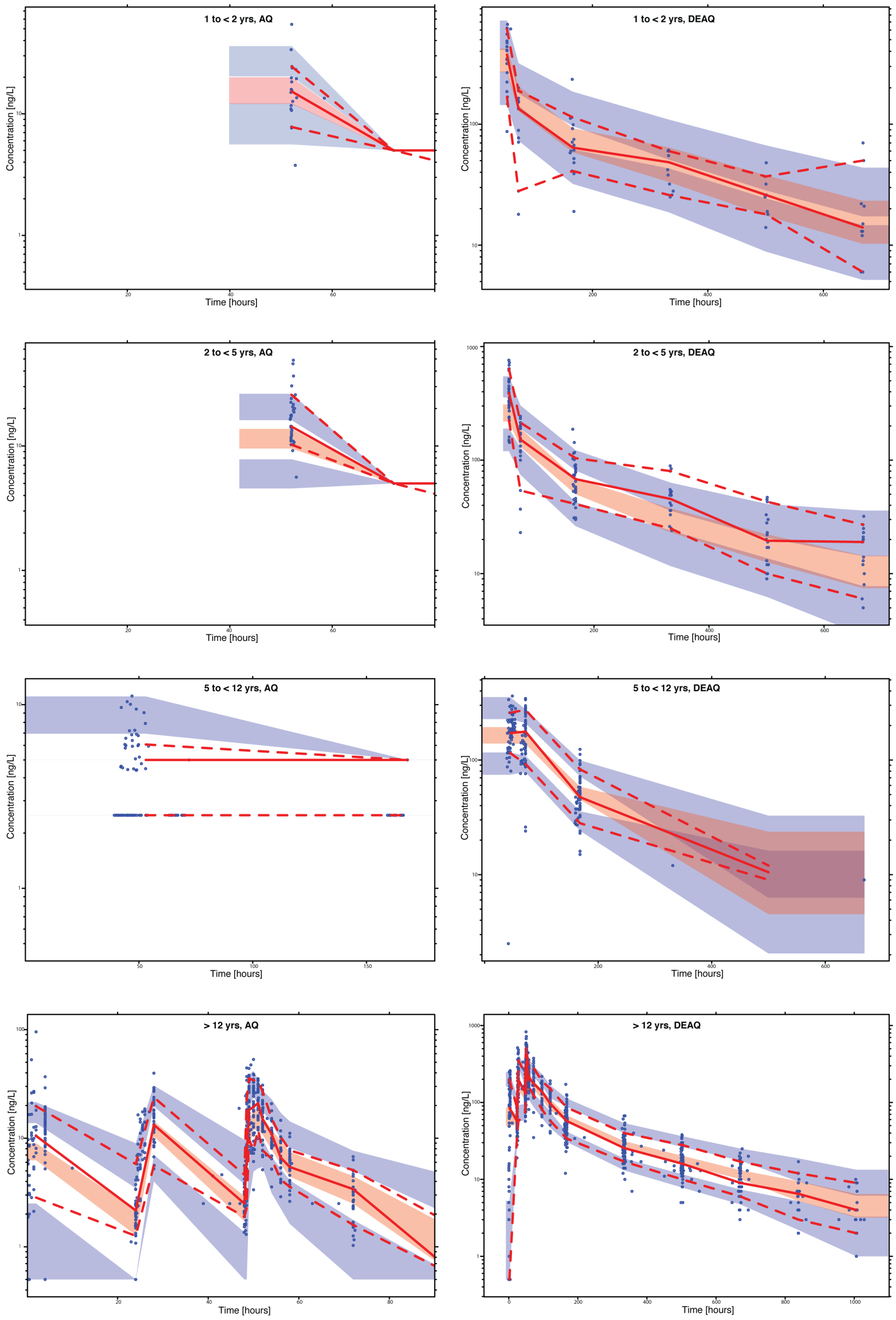

Figure S1. Visual predictive check of the final model describing the plasma concentrations of amodiaquine (AQ, left panels) and desethylamodiaquine (DEAQ, right panels) vs. time, stratified according to age groups. Open circles are the observed data points; solid and dashed lines are the 50th, 5th, and 95th percentiles of the observed data; shaded areas are the simulated (n=1000) 95% confidence intervals for the same percentiles. Plasma concentrations are shown on the log-scale. All observed and simulated values below the LLOQ have been imputed to LLOQ/2, using the LLOQ value specific to the study that contributed each observation. This is the reason for the horizontal percentile lines and thin confidence intervals visible in the panels referring to the lowest age groups. The VPC in the age group > 12 years for AQ was cut at 90 hours after the first dose since beyond this time the concentration of both observed and simulated data were below the LLOQ.

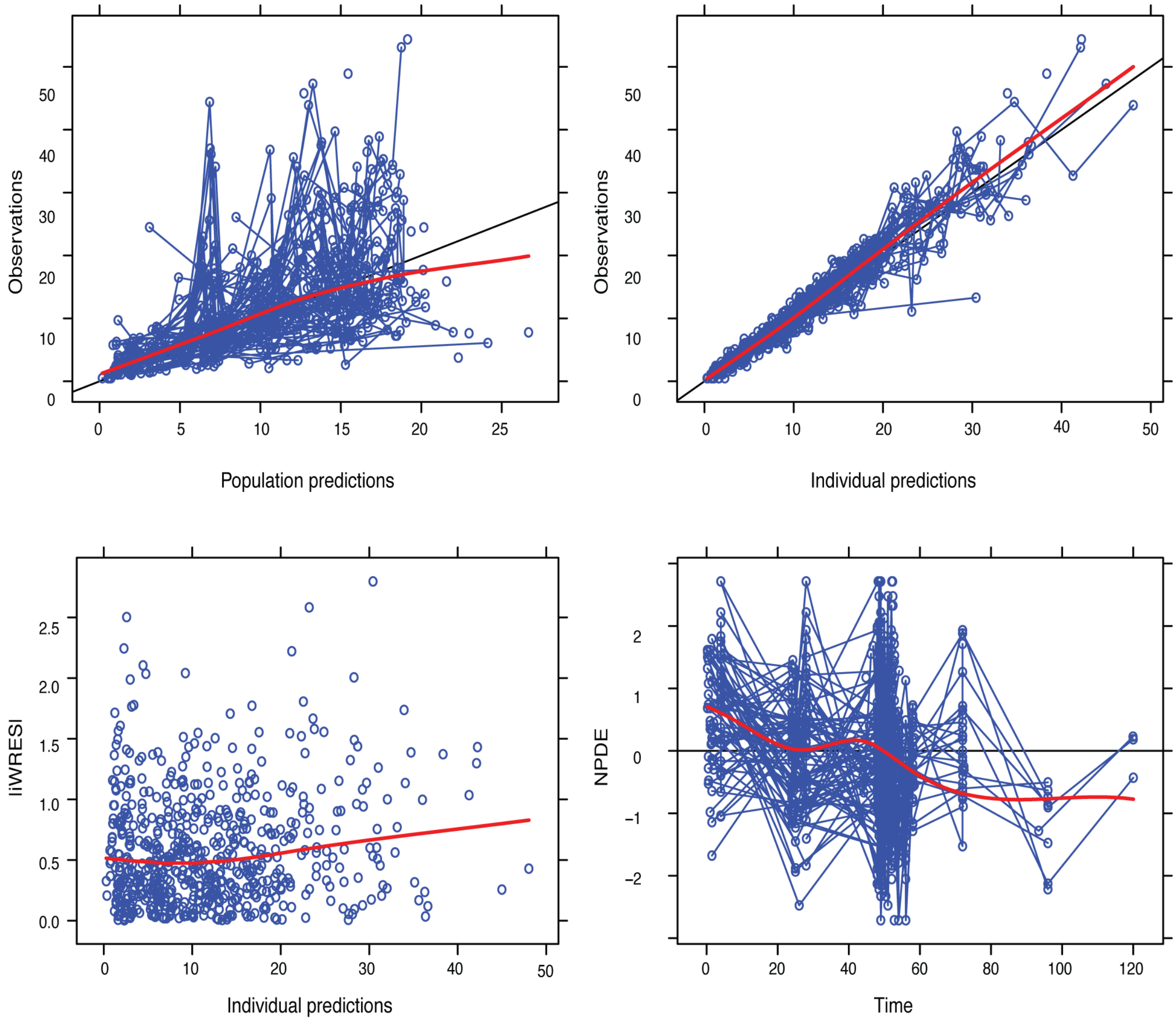

Figure S2A. Basic goodness-of-fit diagnostic of the final population pharmacokinetic model of amodiaquine in uncomplicated P. falciparum malaria patients. Red lines, locally weighted least-squares regression; black lines, line of identity.

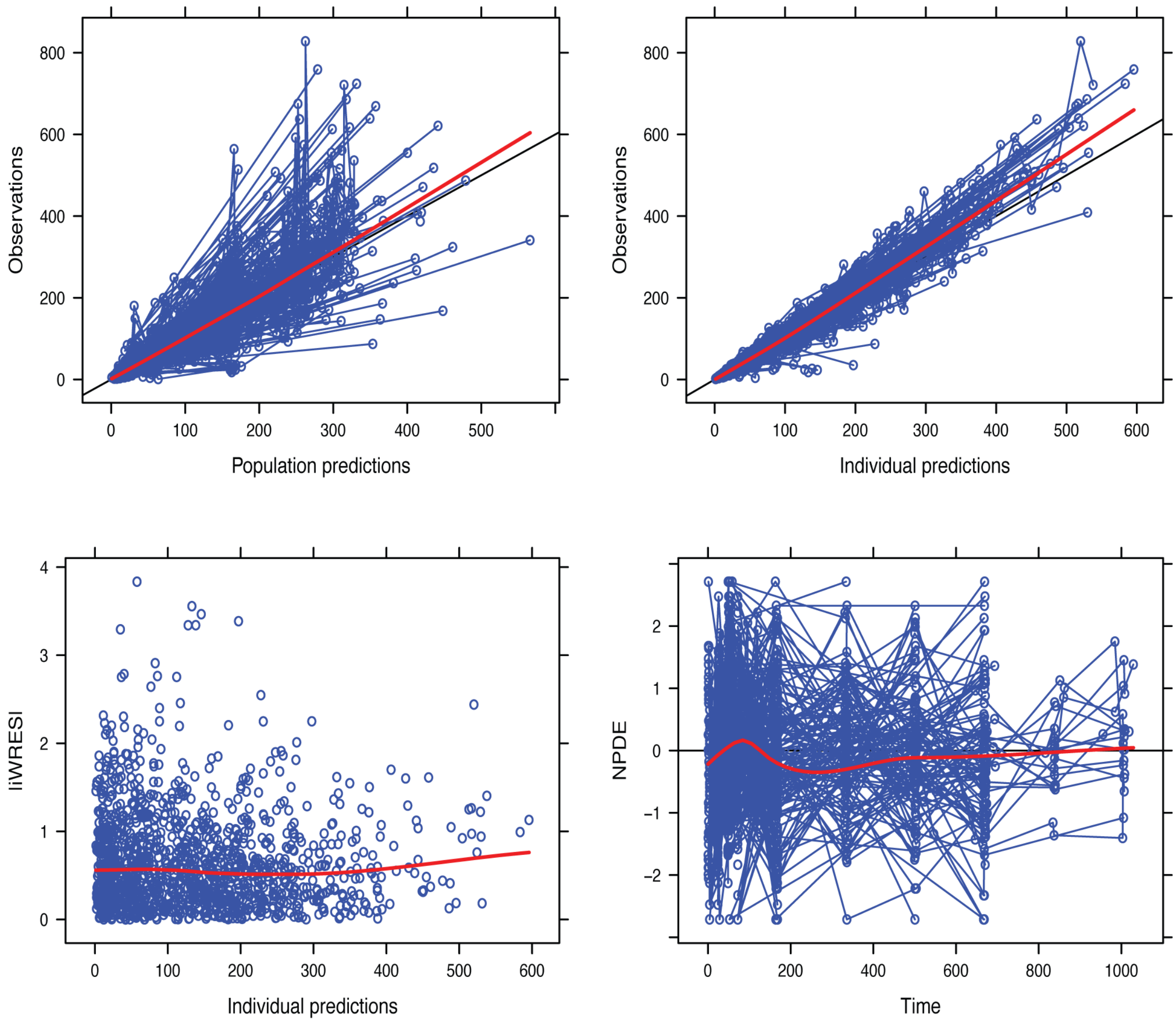

Figure S2B. Basic goodness-of-fit diagnostic of the final population pharmacokinetic model of desethylamodiaquine in uncomplicated P. falciparum malaria patients. Red lines, locally weighted least-squares regression; black lines, line of identity.
